# Precise estimation of human corticospinal excitability associated with the levels of motor imagery-related EEG desynchronization extracted by a locked-in amplifier algorithm

**DOI:** 10.1186/s12984-018-0440-5

**Published:** 2018-11-01

**Authors:** Kensho Takahashi, Kenji Kato, Nobuaki Mizuguchi, Junichi Ushiba

**Affiliations:** 1 Faculty of Science and Technology, Keio University, 3-14-1 Hiyoshi, Kohoku-ku, Yokohama, Kanagawa 223-8522 Japan; 2 Department of Rehabilitation Medicine, Keio University School of Medicine, 35 Shinanomachi, Shinjuku, Tokyo, 160-8582 Japan; 3 The Japan Society for the Promotion of Science, 5-3-1 Kojimachi, Chiyoda-ku, Tokyo, 102-0083 Japan; 4 Keio Institute of Pure and Applied Sciences (KiPAS), Keio University, 3-14-1 Hiyoshi, Kohoku-ku, Yokohama, Kanagawa 223-8522 Japan; 5 Department of Biosciences and Informatics, Faculty of Science and Technology, Keio University, 3-14-1 Hiyoshi, Kouhoku-ku, Yokohama, Kanagawa 223-8522 Japan; 6 Present address: Center of Assistive Robotics and Rehabilitation for Longevity and Good Health, National Center for Geriatrics and Gerontology, 7-430, Morioka-cho, Obu, Aichi 474-8511 Japan

**Keywords:** Brain-computer interface (BCI), Transcranial magnetic stimulation (TMS), Corticospinal excitability, Event-related desynchronization (ERD), Electroencephalogram (EEG), Sensorimotor cortex (SM1)

## Abstract

**Background:**

Physical motor exercise aided by an electroencephalogram (EEG)-based brain-computer interface (BCI) is known to improve motor recovery in patients with stroke. In such a BCI paradigm, event-related desynchronization (ERD) in the alpha and beta bands extracted from EEG recorded over the primary sensorimotor area (SM1) is often used, since ERD has been suggested to be associated with an increase of corticospinal excitability. Recently, we demonstrated a novel online lock-in amplifier (LIA) algorithm to estimate the amplitude modulation of motor-related SM1 ERD. With this algorithm, the delay time, accuracy, and stability to estimate motor-related SM1 ERD were significantly improved compared with the conventional fast Fourier transformation (FFT) algorithm. These technical improvements to extract an ERD trace imply a potential advantage for a better trace of the excitatory status of the SM1 in a BCI context. Therefore, the aim of this study was to assess the precision of LIA-based ERD tracking for estimation of corticospinal excitability using a transcranial magnetic stimulation (TMS) paradigm.

**Methods:**

The motor evoked potentials (MEPs) induced by single-pulse TMS over the primary motor cortex depending on the magnitudes of SM1 ERD (i.e., 35% and 70%) extracted by the online LIA or FFT algorithm were monitored during a motor imagery task of wrist extension in 17 healthy participants. Then, the peak-to-peak amplitudes of MEPs and their variabilities were assessed to investigate the precision of the algorithms.

**Results:**

We found greater MEP amplitude evoked by single-pulse TMS triggered by motor imagery-related alpha SM1 ERD than at rest. This enhancement was associated with the magnitude of ERD in both FFT and LIA algorithms. Moreover, we found that the variabilities of peak-to-peak MEP amplitudes at 35% and 70% ERDs calculated by the novel online LIA algorithm were smaller than those extracted using the conventional FFT algorithm.

**Conclusions:**

The present study demonstrated that the calculation of motor imagery-related SM1 ERDs using the novel online LIA algorithm led to a more precise estimation of corticospinal excitability than when the ordinary FFT-based algorithm was used.

**Electronic supplementary material:**

The online version of this article (10.1186/s12984-018-0440-5) contains supplementary material, which is available to authorized users.

## Background

Physical motor exercise aided by an electroencephalogram (EEG)-based brain-computer interface (BCI) facilitates functional recovery in patients with motor deficits due to stroke [1–12]. In such a BCI paradigm, event-related desynchronization (ERD) in the alpha (8–13 Hz) and beta (15–30 Hz) bands is extracted from EEG signals recorded over the primary sensorimotor area (SM1), and visual and sensory feedback contingent to the extent of ERD is provided via a motor-driven orthosis or neuromuscular electrical stimulation. The repetition of BCI-aided motor exercise in finger opening or arm reaching paradigms is suggested to help patients learn to activate sensorimotor cortical neurons more efficiently than exercises without a BCI [9, 13].

Previous studies using transcranial magnetic stimulation (TMS) delivered to the primary motor cortex have shown that an increase of corticospinal excitability is associated with ERD [14] and is accompanied with a decrease of GABAergic intracortical inhibition [15], indicating ERD as a biomarker of corticospinal excitability. However, the real-time estimation of ERD trace results has a time delay measured in hundreds of milliseconds. A variety of spectral analyses, such as fast Fourier transformation (FFT) [8, 10, 12, 16–20], continuous Wavelet transformation [21, 22], an autoregressive model [9, 23, 33], have been used to calculate the frequency spectrum in a given time-sliding window with certain overlaps, but the results were smoothed due to window overlapping, and were delayed due to window length, causing the inevitable limited resolution of ERD.

Recently, we successfully developed a novel online lock-in amplifier (LIA) algorithm to estimate the amplitude modulation of motor imagery-related alpha ERD over SM1 [24]. LIA can reliably extract signal amplitude in a defined frequency band using a point-by point multiplication and filtering algorithms. Using this algorithm, the delay time, accuracy, and stability to estimate motor-related SM1 ERD were significantly improved compared with those calculated by the conventional online FFT, continuous Wavelet transformation, and autoregressive algorithms. These technical improvements to extract an ERD trace imply a potential advantage for a better trace of the excitatory status of SM1 in the context of a BCI.

Therefore, in this paper, we assessed the precision of LIA-based ERD tracking for estimation of corticospinal excitability using a TMS paradigm. We monitored the motor evoked potentials (MEPs) induced by single-pulse TMS over the primary motor cortex depending on the magnitude of SM1 ERD (i.e., 35% and 70%) extracted by the novel online LIA and conventional FFT algorithms during a motor imagery task of wrist extension in healthy participants. Then, we compared the peak-to-peak amplitudes of MEPs and their variabilities triggered by the different magnitudes of ERD extracted by the novel LIA and conventional FFT algorithms to investigate their precision of corticospinal excitability.

## Methods

### Participants

Seventeen healthy participants (average age, 23.8 ± 2.8 years) participated in this study. All participants were right-handed, without any medical or psychological disorders according to self-reports. Informed consent was given by all participants after they received an explanation of the experimental procedure. The experimental protocol used in this study was in accordance with the Helsinki Declaration and was approved by the ethics committee of Keio University.

### Data acquisition

#### EEG recordings

EEG signals were recorded with 128-channel Geodesic Sensor Nets over the whole scalp. Electrode impedance was kept lower than 40 kΩ throughout the experiments [25]. The EEG signals were amplified and band-pass and notch filtered between 5 and 70 Hz by the Geodesic EEG System (Electrical Geodesics Incorporated [EGI], Oregon, USA) and then recorded at a sampling rate of 1000 Hz. The EEG signals were extracted using a large Laplacian filter centered on C3, which was defined as the nearest channel to the contralateral SM1 [24], since we recorded EEG sensorimotor responses during motor imagery tasks of right wrist extension (see *Experimental procedures* in the Methods section).

#### ERD estimation algorithms

First, we used an FFT algorithm as the conventional method to probe the motor-imagery-related ERDs [8, 10, 12, 15, 19, 20]. According to these previous studies, EEG data were processed using the following 4 steps: (1) segmentation of 1-s time windows with 99% overlap; (2) power spectrum density calculation by FFT algorithm with a Hanning window; (3) determination of a frequency of interest (FOI), which showed the most significant ERD over the alpha bands by visual inspection in Screening session (see *Experimental procedures* in the Methods section); and (4) ERD transformation [15]. The algorithm of the motor-related ERD was defined as follows:

$$ \mathrm{ERD}\left(f,t\right)=\frac{A\left(f,t\right)-R(f)}{R(f)}\times 100 $$

where *A* is the power spectrum density (PSD) of the EEG signal and *R* is the PSD of the baseline period from 3 to 5 s in each resting phase at time *t* and frequency *f*, which was most reactive frequency displaying ERD over the alpha band during the kinesthetic motor imagery task in the screening session.

Second, we used LIA-based algorithm as a novel estimation method [24]. According to the previously established algorithm with LIA [24], EEG data were processed using the following 4 steps: (1) determination of a FOI; (2) applying a narrow band-pass filter (FOI ± 1 Hz) using a second-order Butterworth band-pass filter with a 1-s time window; (3) LIA process (i.e. point-by-point multiplication and integration of the input signal with a reference trigonometric basis signal) with segmentation of 1/FOI s with a 99% overlap time window; and (4) ERD transformation [15].

In briefly, the previous study suggested that the averaged time delays by the online LIA algorithm (200 ± 9.49 ms) were around 300 ms shorter than those by the online FFT algorithm (503 ± 18 ms) [24]. In addition, the accuracy and stability to detect amplitude modulation of motor-imagery-related ERDs by the online LIA algorithm were significantly higher than those calculated by the online FFT algorithm (*p* < 1.0*10–10, p < 1.0*10–9, respectively) [24].

#### Surface electromyography (EMG) recordings

Surface EMG activity was recorded with Ag/AgCl electrodes (fixed electrode distance: 20 mm) over the muscle belly of the right flexor carpi radialis and extensor carpi radialis (ECR) muscles. Impedance for all channels was maintained below 20 kΩ through the experiments. EMG signals were band-pass filtered (5–1000 Hz with 2nd order Butterworth) with a notch (50 Hz to avoid power line contamination), and digitized at 2 kHz using a bio-signal amplifier (Neuropack MEB-9200; Nihon Kohden, Tokyo, Japan). The pre-stimulus EMG activity and MEP were analyzed using − 50 to 150 ms periods of each pulse in an offline process.

#### TMS protocol

Single-pulse TMS was applied with a double-cone coil (outer diameter of each coil: 11 cm, angle of each coil: 95°) connected to a Magstim 200 magnetic stimulator (Magstim, Whitland, UK). The optimal coil position was determined where the motor evoked potential (MEP) amplitude in the ECR was observed with the lowest stimulus intensity and marked with the Brainsight TMS navigation system (Rogue Research, Cardiff, UK). The optimal coil orientation and location remained constant throughout the session. The resting motor threshold (rMT) intensity was defined as the lowest stimulator output intensity capable of inducing an MEP with at least 50 μV peak-to-peak amplitude in relaxed muscles in at least half of the 10 trials [26, 27]. TMS in all experiments was applied with an intensity of 120% of the individual rMT. The values of rMT in each participant are shown in Table 1. We inspected the EMG data during offline analysis, discarding any trials containing pre-stimulus EMG activities more than ±20 μV. Less than 5% of all trials were rejected due to contamination.

**Table 1:** Most reactive frequency displaying ERD during the right wrist motor imagery task in the screening session, resting motor threshold, and stimulus intensity in each participant

Participant	Age (years)	Frequency (Hz)	Resting motor threshold (%MSO)	Stimulus intensity (%MSO)
1	23	12	43	52
2	21	9	45	54
3	21	13	36	43
4	26	13	43	52
5	24	11	38	46
6	24	13	43	52
7	21	13	33	40
8	23	9	36	43
9	28	12	37	44
10	31	10	42	50
11	24	13	32	38
12	23	12	44	53
13	26	13	41	49
14	21	12	44	53
15	22	12	43	52
16	26	13	39	47
17	22	9	39	47

*MSO* maximum stimulator output

### Experimental procedures

#### Screening session

**Fig. 1:**
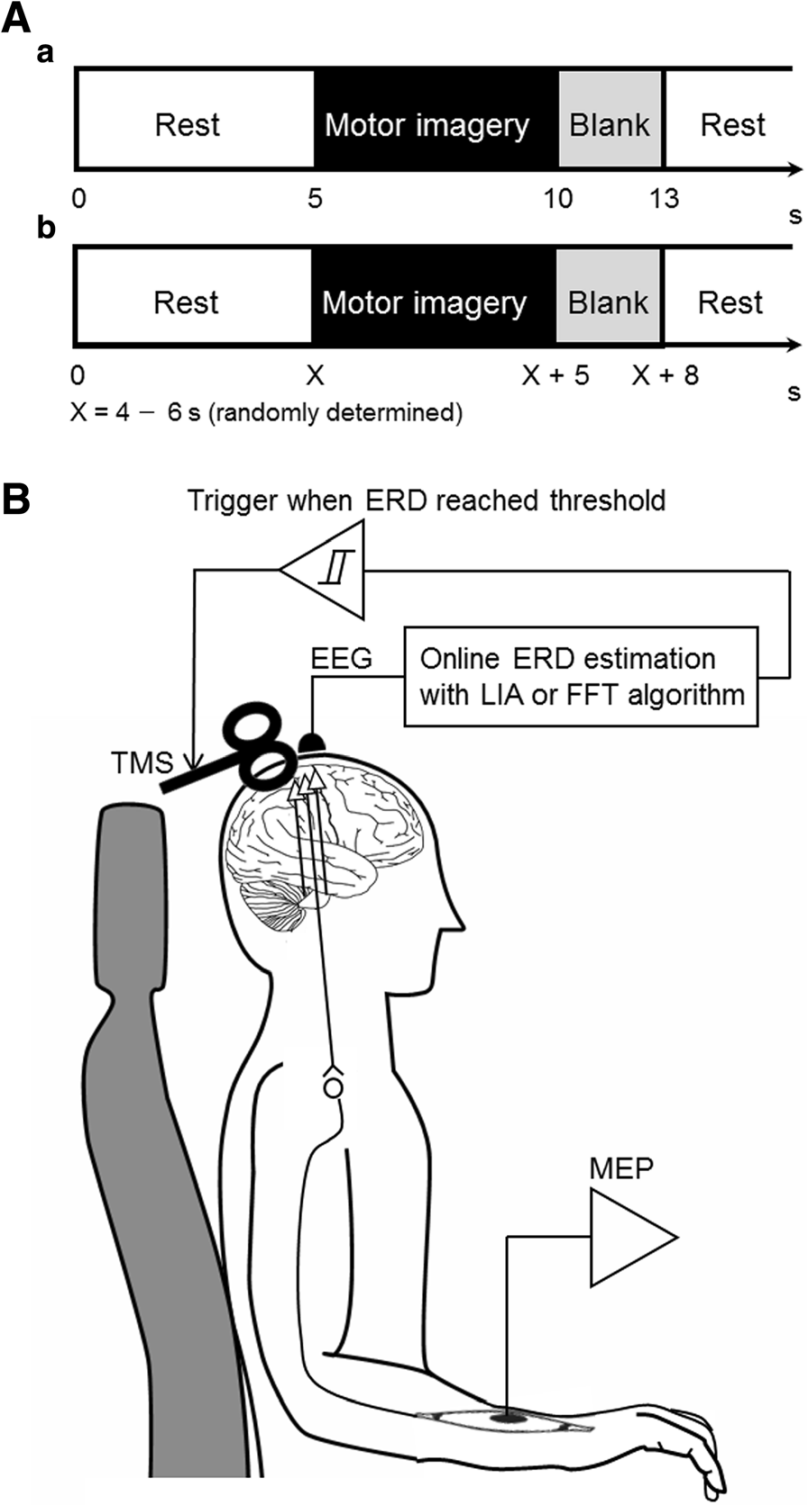
Task protocol and experimental setup. **A**: Timing of the paradigm used in the screening session (**a**) and TMS sessions in *Conditions 2–5* (**b**). **B**: Experimental system of TMS sessions in *Conditions 2–5*. In brief, TMS was applied immediately after instantaneous ERDs calculated by the online FFT or LIA algorithm reached 35% or 70% during motor imagery tasks. ERD, event-related desynchronization; FFT, fast Fourier transformation; LIA; lock-in amplifier; MEP, motor evoked potential; TMS, transcranial magnetic stimulation

Firstly, a screening session was performed to investigate the most reactive frequency associated with the motor imagery-related SM1 ERD in each participant. The participants sat in a comfortable armchair and performed kinesthetic motor imagery of right wrist extension with a fixed repetitive time scheme (Fig. 1Aa). A 20-in. computer monitor was placed 60–90 cm in front of their eyes. Then, the screening session started with the presentation of the word “Rest” at the center of the monitor. After 5 s, the word displayed in the monitor changed to “Image,” and the participant was asked to perform kinesthetic motor imagery of wrist extension for 5 s. Then, the monitor went black and the participant could move freely for 3 s. This overall process was repeated for a total of 25 trials.

#### TMS sessions

Each participant performed a total of 5 experimental conditions with TMS in a predetermined randomized order, which was counterbalanced across the participants. In each condition, the rest period was randomized from 4 to 6 s to prevent the participants from predicting task onset in each trial. Then, TMS was applied to the SM1 at different timings over the 5-s task period depending on the following 5 conditions.

In *Condition 1 “Relaxed”*, the participants were instructed to be relaxed during the task phase instead of performing the motor imagery task, as a control session. TMS was applied randomly during the task phase. The stimulus was applied 25 times.

In *Condition 2 “FFT, ERD35%”* and *Condition 3 “FFT, ERD70%”*, the participants were instructed to perform the kinesthetic motor imagery task of right wrist extension during the task phase. TMS was applied immediately after instantaneous ERDs calculated by the online FFT algorithm reached 35% in *Condition 2* or 70% in *Condition 3* during the motor imagery task. The participants were asked to continue the kinesthetic motor imagery task even after TMS was applied. We ended the session when the number of successful trials (i.e. ERD values were reached to 35% in *Condition 2* or 70% in *Condition 3*) reached 25. In case of trials that the ERD could not be reached to the targeted values, it was not counted as the number of successful trials (i.e. TMS was not applied). The parameters for the time window and overlap used for the FFT algorithm were determined as 1000 ms and 90%, according to the previous study [15].

In *Condition 4 “LIA, ERD35%”* and *Condition 5 “LIA, ERD70%,”* the participants were instructed to perform the same kinesthetic motor imagery task of right wrist extension during the task phase. TMS was applied immediately after instantaneous ERDs calculated by the online LIA algorithm reached 35% in *Condition 4* or 70% in *Condition 5* during the kinesthetic motor imagery task. The participants were asked to continue the kinesthetic motor imagery task even after TMS was applied. We ended the session when the number of successful trials (i.e. ERD values were reached to 35% in *Condition 4* or 70% in *Condition 5*) reached 25. In case of trials that the ERD could not be reached to the targeted values, it was not counted as the number of successful trials (i.e. TMS was not applied).

To reduce the participants’ bias, the order of the conditions 2–5 were blinded for the participants.

### Data analysis and statistics

After removing the trials in which the pre-stimulus EMG activity was more than ±20 μV, the peak-to-peak MEP amplitudes were calculated in each trial and TMS session. Then, the peak-to-peak MEP amplitudes at ERD 35% and ERD 70% (*Conditions 2–5*) were normalized by those at the resting condition (*Condition 1*). Finally, the average and standard deviation (SD) of normalized peak-to-peak MEP amplitudes across trials in every condition and participant were calculated.

The average of normalized peak-to-peak MEP amplitudes across participants was compared between *Condition 1* and *Conditions 2–5* using a t-test, followed with Bonferroni correction. In addition, two-way repeated measures analysis of variance (ANOVA) was performed to compare normalized peak-to-peak amplitude between the 4 TMS conditions (*Conditions 2–5*). The SD of normalized peak-to-peak amplitude was also compared between the four TMS conditions (*Conditions 2–5*) using two-way repeated measures ANOVA. If ANOVA yielded a significant F value, a post-hoc test was then performed using a t-test with Bonferroni correction.

## Results

### Motor imagery-related SM1 ERD

**Fig. 2:**
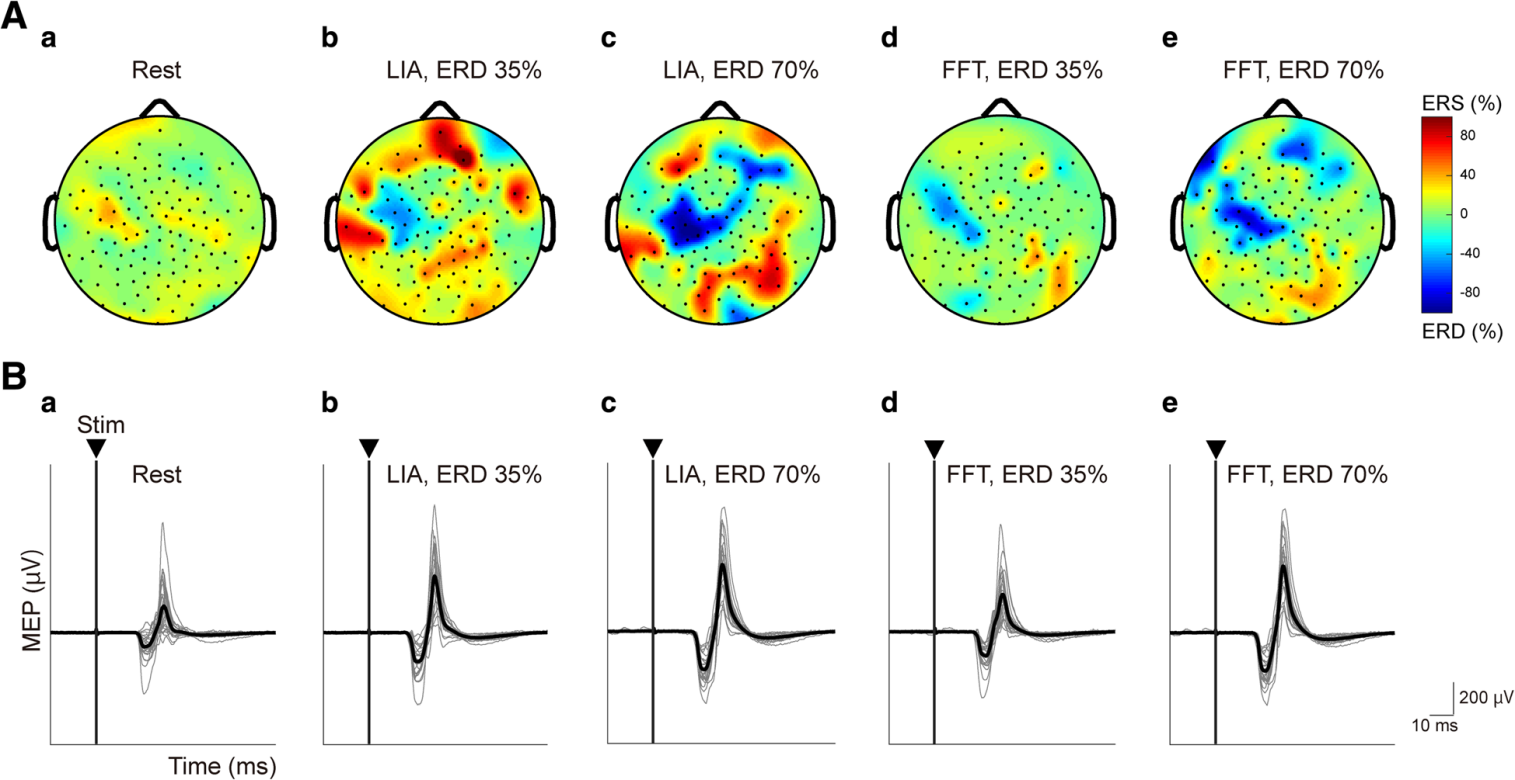
Representative topographic map and MEP traces induced by single-pulse TMS recorded from the right ECR at ERD 35% and ERD 70%, calculated by the online FFT or LIA algorithm. **A**: Representative topographies of the averaged ERDs over trials during the resting condition (**a**), wrist kinesthetic motor imagery at ERD 35% (**b**) and ERD 70% (**c**) calculated by the online FFT algorithm, and ERD 35% (**d**) and ERD 70% (**e**) calculated by the online LIA algorithm, obtained from 128-channel EEG data. Topographic maps are illustrated in the most reactive frequency displaying motor imagery-related ERD. Data were obtained from Subject 9. Electrode positions are shown by dots. Negative values (blue colors) indicate strong ERD. **B**: Example MEP traces induced by single-pulse TMS recorded from the ECR during the resting condition (**a**) and during kinesthetic motor imagery of right wrist extension at ERD 35% (**b**) and ERD 70% (**c**) calculated by the online FFT algorithm, and ERD 35% (**d**) and ERD 70% (**e**) calculated by the online LIA algorithm. Thin gray lines represent representative MEP traces across 25 trials. Thick black lines represent the averaged MEP traces. The triangles and vertical lines represent stimulus onset (Stim). As ERD increased, MEP amplitudes induced by single-pulse TMS were facilitated in both cases of ERD estimations based on the online FFT and LIA algorithms. ECR, extensor carpi radialis; ERD, event-related desynchronization; FFT, fast Fourier transformation; LIA; lock-in amplifier; MEP, motor evoked potential; TMS; transcranial magnetic stimulation. Data were obtained from Subject 9

In the screening session, all participants showed significant task-related alpha ERDs around C3 during the kinesthetic motor imagery task of right wrist extension. The characteristics of the most reactive frequency of motor imagery-related SM1 ERDs are summarized in Table 1. The representative data (Subject 9) of topographic maps of the averaged motor imagery-related SM1 ERDs over 25 trials obtained from 128-channel EEG signals over the most reactive frequency were further shown in *Conditions 1–5* during rest (Fig. 2Aa) and when the ERDs reached 35% and 70% calculated by the online FFT and LIA algorithms during the kinesthetic motor imagery task (Fig. 2Ab-e). Motor imagery-related alpha ERD magnitudes occurred around C3 in *Conditions 2–5*, suggesting that the observed alpha ERD was likely to localize to the contralateral SM1. In addition, one-way ANOVA was performed to compare power values during the resting periods between *Conditions 2–5*. As a result, there was no significant difference in the resting-state power values over the most reactive frequency (F = 1.80, *p* = 0.158), indicating that the reference power values for calculating ERDs did not differ between *Conditions 2–5*. The numbers of unsuccessful trial were less than 4 trials for all conditions.

### Changes in MEP amplitudes at the different levels of ERD magnitudes obtained by the LIA and FFT algorithms

To test corticospinal excitability at certain magnitudes of SM1 ERD during the right wrist kinesthetic motor imagery task, we applied single-pulse TMS over the primary motor cortex during the rest condition (*Condition 1 “Relaxed”*) and during wrist kinesthetic motor imagery at ERD 35% and 70% calculated by the conventional online FFT algorithm (*Condition 2 “FFT, ERD35%”* and *Condition 3 “FFT, ERD70%”*), and by the novel online LIA algorithm (*Condition 4 “LIA, ERD35%”* and *Condition 5 “LIA, ERD70%”*). Then, we assessed the peak-to-peak MEP amplitudes in each condition.

Representative waveforms of MEPs in the right ECR muscle during the relaxed (*Condition 1*) and kinesthetic motor imagery (*Conditions 2–5*) tasks in a single participant (Subject 9) are shown in Fig. 2B. MEP amplitudes evoked by single-pulse TMS were facilitated during kinesthetic motor imagery (rest = 195.1 ± 106.1 μV, motor imagery at ERD 35% by FFT and LIA =353.3 ± 189.9 and 640.6 ± 163.0 μV, and motor imagery at ERD 70% by FFT and LIA = 638.8 ± 250.7 and 730.1 ± 144.1 μV, respectively). The averaged peak-to-peak MEP amplitudes induced by single-pulse TMS from the ECR across participants were compared between each condition. As a result, the averaged MEP amplitudes were significantly greater in *Condition 2 “FFT, ERD35%”* (*p* < 0.001), *Condition 3 “FFT, ERD70%”* (*p* < 0.05), *Condition 4 “LIA, ERD35%”* (*p* < 0.05), and *Condition 5 “LIA, ERD70%”* (*p* < 0.001), compared to *Condition 1 “Relaxed”* (Additional file 1: Figure S1), indicating that corticospinal excitability was increased more when motor imagery-related alpha SM1 ERD occurred than during the resting state, irrespective of ERD magnitude (i.e., 35% or 70%) and estimation algorithm (i.e., online LIA or FFT algorithm). On the other hand, there were no significant differences in the averaged MEP amplitudes between *Condition 2 “FFT, ERD35%”* and *Condition 4 “LIA, ERD35%”* (*p* = 0.655), and between *Condition 3 “FFT, ERD70%”* and *Condition 5 “LIA, ERD70%”* (*p* = 0.661), suggesting that the averaged MEP amplitudes did not influence to estimation algorithms in each ERD magnitude.

**Fig. 3:**
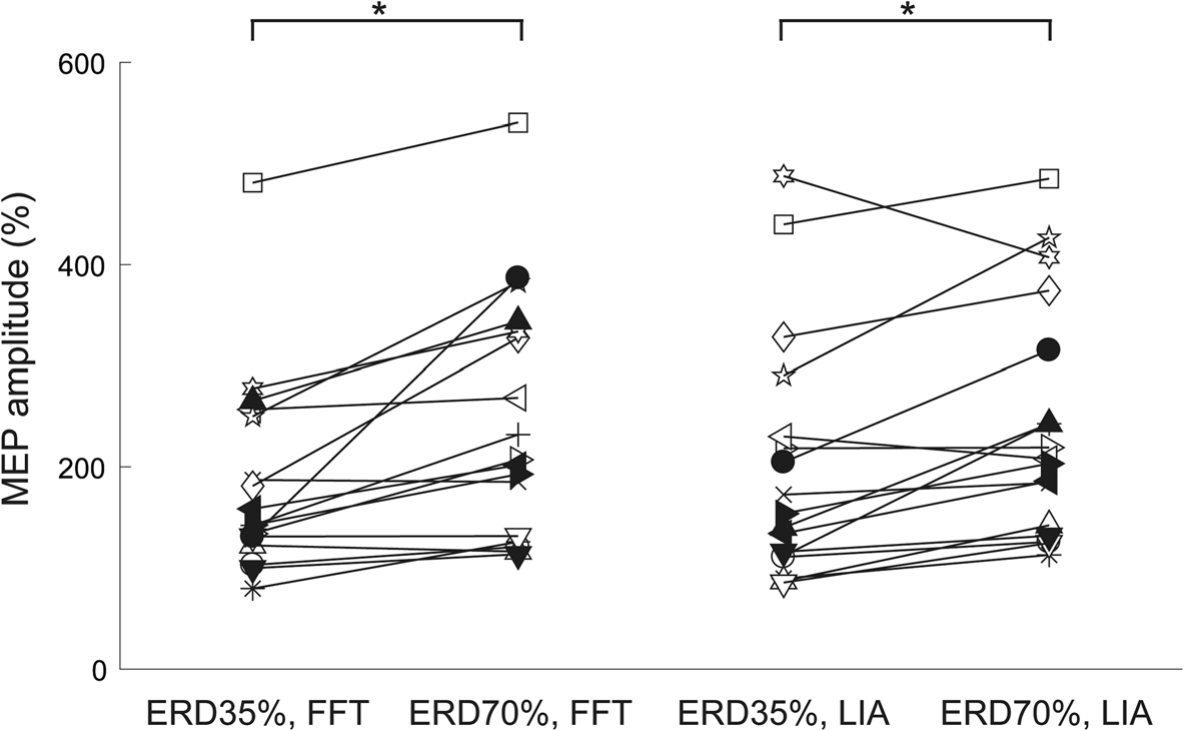
Normalized peak-to-peak MEP amplitudes during motor imagery of right wrist extension at ERD 35% and ERD 70%, calculated by the online FFT or LIA algorithm. The averaged normalized peak-to-peak MEP amplitudes in *Condition 3 “FFT, ERD70%”* showed a significant increase compared to those in *Condition 2 “FFT, ERD35%.”* Similarly, the averaged MEP amplitudes in *Condition 5 “LIA, ERD70%”* showed a significant increase compared to those in *Condition 4 “LIA, ERD35%,”* suggesting that in both online estimation algorithms (FFT and LIA), SM1 excitability was significantly associated with the different levels of motor imagery-related alpha ERDs. Each line shows the result obtained from each participant. *p < 0.05. ERD, event-related desynchronization; FFT, fast Fourier transformation; LIA, lock-in amplifier; MEP, motor evoked potential

Next, normalized peak-to-peak MEP amplitudes were compared between the four ERD-triggered TMS conditions (*Conditions 2–5*) with two-way ANOVA (Fig. 3), indicating the main effect of ERD values (F (1, 14) = 16.51, *p* < 0.01). A post-hoc test using a t-test with Bonferroni correction further suggested that the normalized MEP amplitudes in *Condition 3 “FFT, ERD70%”* showed a significant increase compared to those in *Condition 2 “FFT, ERD35%”* (post hoc *p* < 0.001). Similarly, the normalized MEP amplitudes in *Condition 5 “LIA, ERD70%”* showed a significant increase compared to those in *Condition 4 “LIA, ERD35%”* (post hoc *p* < 0.001), suggesting that in both online estimation algorithms, SM1 excitability was significantly associated with the different levels of motor imagery-related alpha ERDs. On the other hand, there were no significant differences in the normalized MEP amplitudes between *Condition 2 “FFT, ERD35%”* and *Condition 4 “LIA, ERD35%”* (*p* = 0.922), and between *Condition 3 “FFT, ERD70%”* and *Condition 5 “LIA, ERD70%”* (*p* = 0.583), suggesting that the normalized MEP amplitudes did not influence to estimation algorithms in each ERD magnitude.

### Changes in the variabilities of MEP amplitudes at different levels of ERD magnitudes obtained by the LIA and FFT algorithms

**Fig. 4:**
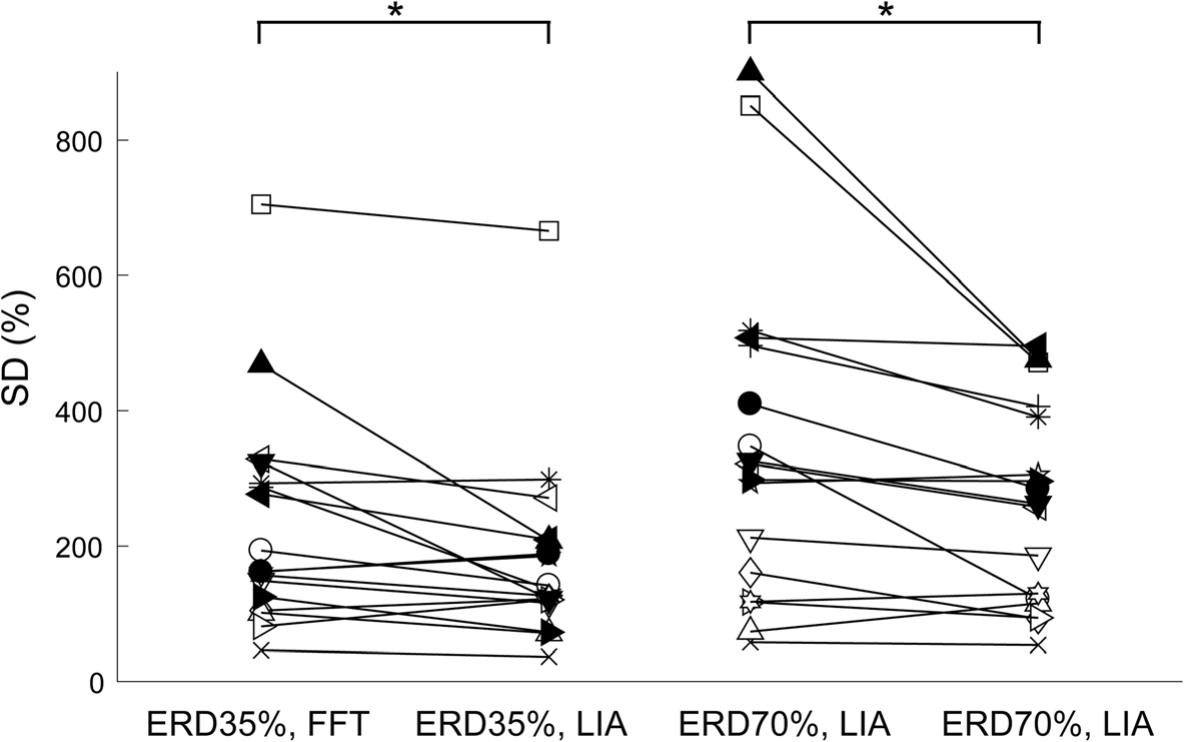
Standard deviations (SDs) of normalized peak-to-peak MEP amplitudes during motor imagery of right wrist extension at ERD 35% and ERD 70%, calculated by the online FFT or LIA algorithm. The SD of normalized peak-to-peak MEP amplitudes in *Condition 4 “LIA, ERD35%”* was smaller than that in *Condition 2 “FFT, ERD35%.”* Similarly, the SD of normalized peak-to-peak MEP amplitudes in *Condition 5 “LIA, ERD70%”* was smaller than that in *Condition 3 “FFT, ERD70%.”* Each line shows the result obtained from each participant. *p < 0.05. ERD, event-related desynchronization; FFT, fast Fourier transformation; LIA; lock-in amplifier; MEP, motor evoked potential

To investigate the estimation precision of instantaneous corticospinal excitability reflected by the MEP amplitudes induced by single-pulse TMS triggered by the level of motor imagery-related ERDs, we assessed the SD of normalized peak-to-peak MEP amplitudes at ERD 35% and ERD 70%, extracted by the conventional online FFT algorithm (*Condition 2 “FFT, ERD35%”* and *Condition 3 “FFT, ERD70%”*) and novel LIA algorithm (*Condition 4 “LIA, ERD35%”* and *Condition 5 “LIA, ERD70%”*), and compared them using two-way ANOVA (Fig. 4). As a result, we found main effects of ERD magnitudes (i.e., ERD 35% and ERD 70%) [F (1, 14) = 6.81, *p* < 0.05] and algorithms (i.e., FFT and LIA algorithms) [F (1, 14) = 5.17, *p* < 0.05]. Moreover, an interaction was found between both factors [F (1, 14) = 8.69, *p* < 0.05]. Post hoc tests with Bonferroni’s correction showed that the SD of normalized peak-to-peak MEP amplitudes in *Condition 4 “LIA, ERD35%”* was smaller than that in *Condition 2 “FFT, ERD35%”* (*p* < 0.05, Fig. 4). Similarly, the SD of normalized peak-to-peak MEP amplitudes in *Condition 5 “LIA, ERD70%”* was smaller than that in *Condition 3 “FFT, ERD70%”* (*p* < 0.05, Fig. 4). These results suggest that the variabilities of MEP amplitudes at both levels of ERD magnitude (i.e., ERD 35% and ERD 70%) extracted by the novel online LIA algorithm were reduced compared to those extracted by the conventional FFT algorithm.

## Discussion

In the present study, we found greater MEP amplitude evoked by single-pulse TMS triggered by motor imagery-related alpha SM1 ERDs than that randomly applied at rest. This enhancement was associated with the magnitude of ERD (i.e., 35% and 70%) for both the FFT and LIA algorithms, which is consistent with a previous study [15]. Moreover, we found that the variabilities of peak-to-peak MEP amplitudes at the levels of ERD (i.e., 35% and 70%) calculated by the novel online LIA algorithm were reduced compared to those extracted by the conventional FFT algorithm. Based on the previous findings that LIA-based algorithm can detect motor-related SM1 ERD more accurately and stably than conventional FFT algorithm [24], the present results suggest that the accurate calculation of motor imagery-related SM1 ERDs extracted by the novel online LIA algorithm led to a precise estimation of human corticospinal excitability.

Extensive previous studies have consistently demonstrated that alpha/beta ERDs following kinesthetic motor imagery are a reliable biomarker for increased excitabilities in the sensorimotor cortex and corticospinal tract [28–31]. Previous TMS studies have shown an inverse correlation between MEP amplitude and alpha band power [32, 33], and an inverse correlation between intracortical inhibition and the alpha ERD level during kinesthetic motor imagery [15]. Indeed, an additional analysis revealed that both algorithms detected targeted values of ERD over SM1 (Additional file 1: Figure S2). In addition, the magnitude of motor imagery-related SM1 ERD during kinesthetic motor imagery was associated with an increase in F-wave persistence, indicating the potentiation of spinal motoneurons during kinesthetic motor imagery accompanied with SM1 ERD [19]. The present findings also reproduced the correlation between the magnitude of motor imagery-related SM1 ERD and MEP amplitude. Moreover, we notably demonstrated the reduced variability of the results when the novel LIA algorithm was used compared to the FFT algorithm. This result suggests that motor imagery-related SM1 ERDs extracted by the LIA algorithm would be a more reliable biomarker to represent increased excitabilities in sensorimotor cortex and corticospinal tract than when an ordinary FFT-based algorithm is used.

A finger movement exercise aided by an ipsilesional SM1 ERD-based BCI stimulates SM1 while attempting paralyzed finger movements, resulting in an improvement of impaired movements [13]. Therefore, the precise estimation of the ERD trace by the newly proposed LIA algorithm and resulting improved correlation between SM1 ERD and peak-to-peak MEP could be potentially more beneficial for neurorehabilitation than the existing algorithm. For instance, delayed visual feedback attenuates error-based sensorimotor learning during prism adaptation [34, 35] and a gradual visuomotor rotation task [36]. Since BCI-mediated movement exercise is characterized by error-based learning through ERD state-dependent visual feedback [13, 20], the LIA-based instantaneous and accurate visual feedback of sensorimotor and corticospinal excitability assures a stable input-output association, resulting in efficient error-based learning. Further studies are awaited to investigate whether BCI learning using the LIA algorithm is more effective for neurorehabilitation than using the conventional method.

In general, lock-in measurements can extract instantaneous amplitude modulation in a defined single frequency by the point-by-point multiplication and integration of an input with a reference trigonometric basis signal [24]. In the present study, the input signal was defined as EEG amplitude modulation over a single frequency, which showed the most reactive ERD during motor imagery. In contrast, previous studies have demonstrated that motor imagery-related ERDs are associated with a broad frequency range including the alpha and beta frequency bands [31, 37, 38]. Although we demonstrated that the magnitudes of ERD calculated over a single frequency are associated with MEP amplitudes, the multi-frequencies calculation by parallel processing of LIAs may allow additional improvements in terms of the precise estimation of corticospinal excitability. In addition, in future study, we need to think to incorporate the other filtering algorithms such as Gaussian, a generalized matched filter to optimize signal noise ratio in real time, or machine learning algorithms to achieve the additional improvements of the LIA-based algorithms [39, 40].

## Conclusion

The present study demonstrated that the variability of normalized peak-to-peak MEP amplitudes at different magnitudes of ERD extracted by a novel LIA algorithm were reduced more than those extracted by the conventional FFT algorithm. This result suggests that the LIA algorithm can be incorporated effectively into existing BCI paradigms to estimate human corticospinal excitability more precisely. This finding would be useful for the neurorehabilitation of patients with a movement disorder.

## Additional file

Additional file 1: **Figure S1.** Peak-to-peak MEP amplitudes during the resting state and motor imagery of right wrist extension at ERD 35% and ERD 70%, calculated by the online FFT or LIA algorithm. The averaged MEP amplitudes were significantly greater in *Condition 2 “FFT, ERD35%”* (*p* < 0.05), *Condition 3 “FFT, ERD70%”* (*p* < 0.001), *Condition 4 “LIA, ERD35%”* (*p* < 0.01), and *Condition 5 “LIA, ERD70%”* (*p* < 0.001), compared to *Condition 1 “Relaxed.”* Each line shows the result obtained from each participant. *p < 0.05, **p < 0.01, ***p < 0.005. ERD, event-related desynchronization; FFT, fast Fourier transformation; LIA; lock-in amplifier; MEP, motor evoked potential. **Figure S2.** Topography map of true positive rate (%) across subjects. True positive rate is defined as a percentage that exceed the targeted ERD value among 25 trials of the motor imagery task. Both the LIA-based and FFT-based methods can specifically detect the motor-imagery-related ERDs from the vicinity of the C3. (ZIP 3206 kb)

## Abbreviations

BCI: brain-computer interface
ECR: extensor carpi radialis
EEG: electroencephalogram
ERD: event-related desynchronization
FFT: fast Fourier transformation
LIA: lock-in amplifier
MEPs: motor evoked potentials
rMT: resting motor threshold
SM1: primary sensorimotor area
TMS: transcranial magnetic stimulation
