# Supplementary figures and images for: Precise estimation of human corticospinal excitability associated with the levels of motor imagery-related EEG desynchronization extracted by a locked-in amplifier algorithm
Source: J Neuroeng Rehabil. 2018 Nov 1;15:93. doi: 10.1186/s12984-018-0440-5 (PMC6211493; doi:10.1186/s12984-018-0440-5)

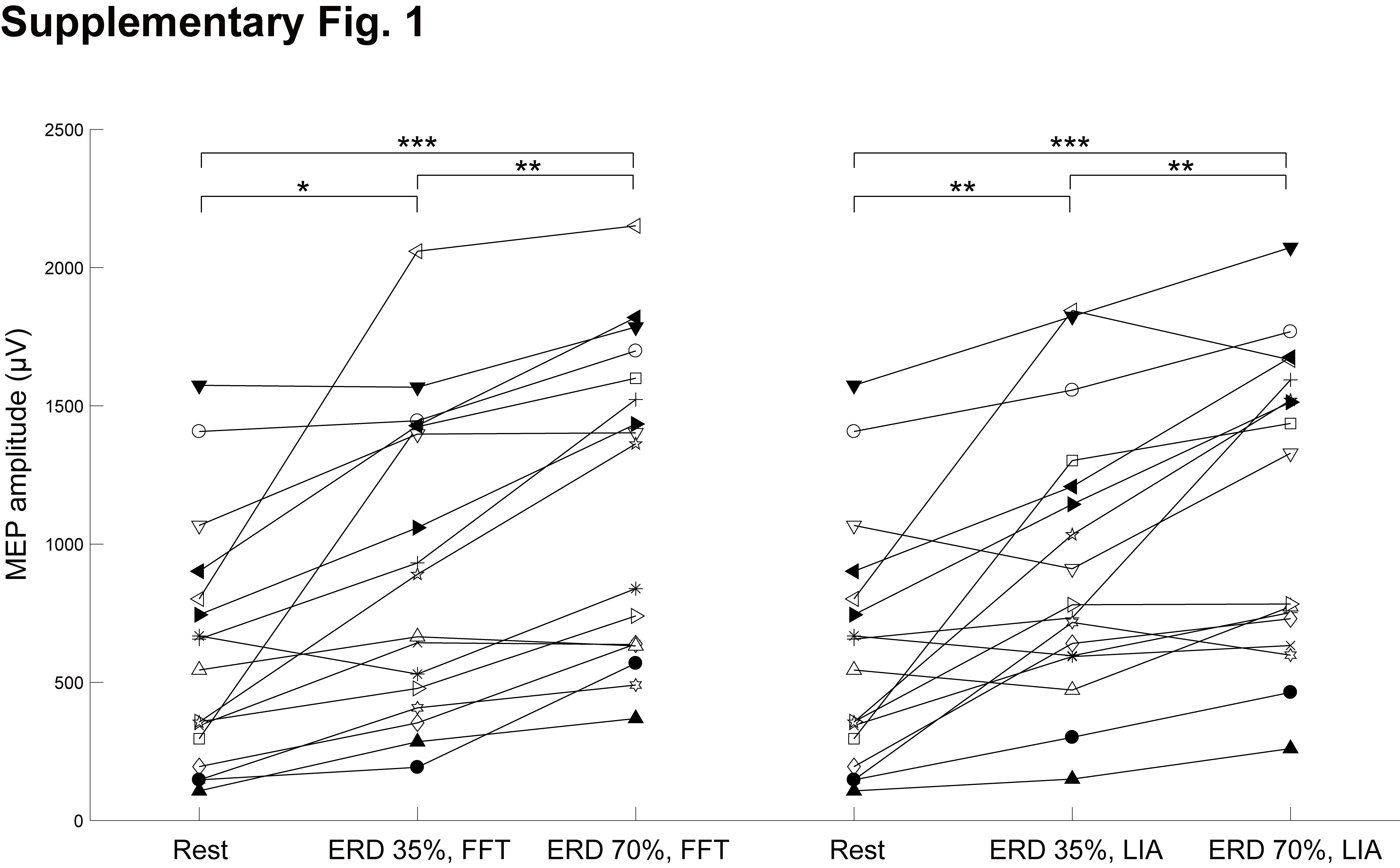

Supplement: Supplementary file 1 — Figure S1. Peak-to-peak MEP amplitudes during the resting state and motor imagery of right wrist extension at ERD 35% and ERD 70%, calculated by the online FFT or LIA algorithm. The averaged MEP amplitudes were significantly greater in Condition 2 “FFT, ERD35%” (p < 0.05), Condition 3 “FFT, ERD70%” (p < 0.001), Condition 4 “LIA, ERD35%” (p < 0.01), and Condition 5 “LIA, ERD70%” (p < 0.001), compared to Condition 1 “Relaxed.” Each line shows the result obtained from each participant. *p < 0.05, **p < 0.01, ***p < 0.005. ERD, event-related desynchronization; FFT, fast Fourier transformation; LIA; lock-in amplifier; MEP, motor evoked potential. Figure S2. Topography map of true positive rate (%) across subjects. True positive rate is defined as a percentage that exceed the targeted ERD value among 25 trials of the motor imagery task. Both the LIA-based and FFT-based methods can specifically detect the motor-imagery-related ERDs from the vicinity of the C3. (ZIP 3206 kb) [file 12984_2018_440_MOESM1_ESM.zip › SupplementaryFig1.tif]

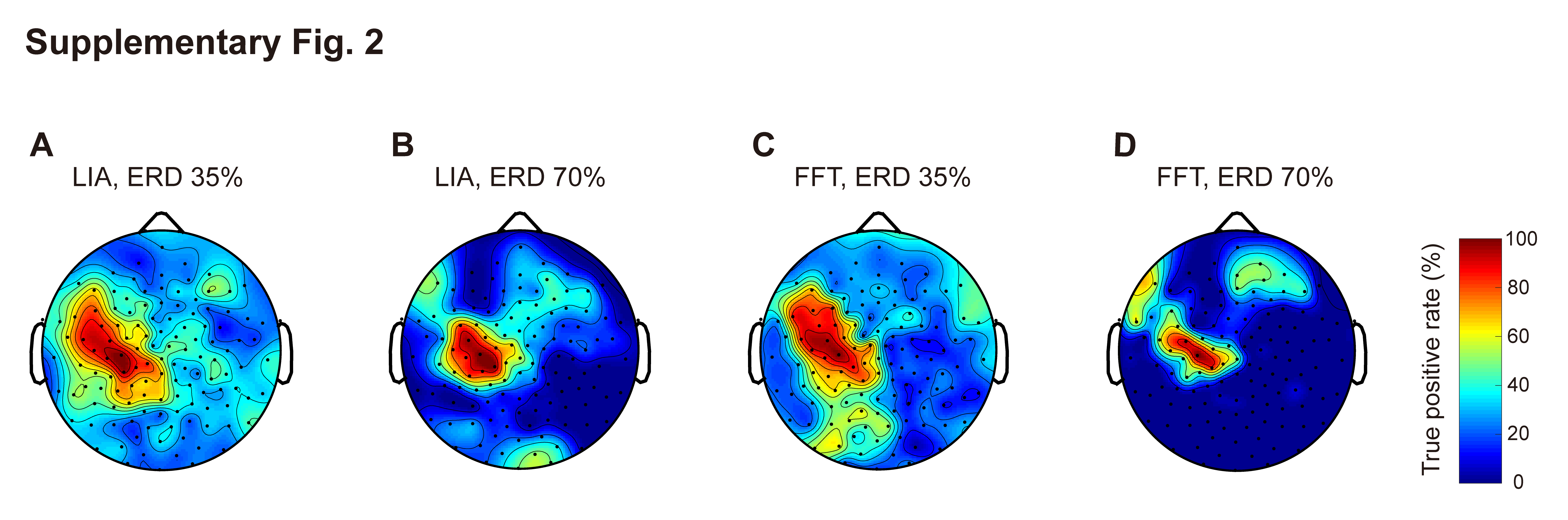

Supplement: Supplementary file 1 — Figure S1. Peak-to-peak MEP amplitudes during the resting state and motor imagery of right wrist extension at ERD 35% and ERD 70%, calculated by the online FFT or LIA algorithm. The averaged MEP amplitudes were significantly greater in Condition 2 “FFT, ERD35%” (p < 0.05), Condition 3 “FFT, ERD70%” (p < 0.001), Condition 4 “LIA, ERD35%” (p < 0.01), and Condition 5 “LIA, ERD70%” (p < 0.001), compared to Condition 1 “Relaxed.” Each line shows the result obtained from each participant. *p < 0.05, **p < 0.01, ***p < 0.005. ERD, event-related desynchronization; FFT, fast Fourier transformation; LIA; lock-in amplifier; MEP, motor evoked potential. Figure S2. Topography map of true positive rate (%) across subjects. True positive rate is defined as a percentage that exceed the targeted ERD value among 25 trials of the motor imagery task. Both the LIA-based and FFT-based methods can specifically detect the motor-imagery-related ERDs from the vicinity of the C3. (ZIP 3206 kb) [file 12984_2018_440_MOESM1_ESM.zip › SupplementaryFig2.tif]
